# Curcumin: A Promising Tool to Develop Preventive and Therapeutic Strategies against Non-Communicable Diseases, Still Requiring Verification by Sound Clinical Trials

**DOI:** 10.3390/nu14071401

**Published:** 2022-03-28

**Authors:** Roberta Masella, Francesca Cirulli

**Affiliations:** 1 Centre for Gender-Specific Medicine, Istituto Superiore di Sanità, Viale Regina Elena 299, 00161 Rome, Italy; 2 Center for Behavioral Sciences and Mental Health, Istituto Superiore di Sanità, Viale Regina Elena 299, 00161 Rome, Italy; francesca.cirulli@iss.it

Curcumin is a pleiotropic compound found in the rhizome of Curcuma longa (turmeric). Curcuma longa has been widely used as a spice for a long time, especially in Asian countries; however, the interest in this compound has been growing and it is largely consumed as dietary component and supplement all around the world. The great interest in curcumin has been due to a number of potential biological activities this compound has shown over time, and it is well documented by the 8601 papers—of which 229 are clinical trials—retrieved in PubMed on 12 January 2022, published in the last five years using ‘curcumin’ as keyword. It is important to underline that, since the majority of such studies were carried out in cellular and animal models, conclusive evidence of the real effectiveness of curcumin as a preventive and therapeutic compound is still far from being reached, although the number of clinical trials addressing the potential therapeutic role of curcumin in a number of pathological and non-pathological conditions is growing every day. It is safe to say that, despite its reported benefits, one of the major drawbacks of ingesting curcumin is its poor bioavailability, thus major efforts are needed to overcome this problem.

The Special Issue, “Dietary Curcumin and Health Effects”, was aimed at collecting the most advanced evidence on the relationship between curcumin and health, with the final objective to improve research and move the field forward.

The Special Issue provides twelve contributions, of which three are original articles, seven are narrative reviews, one is a systematic review and one is a meta-analysis, that all together offer a multifaceted and multidisciplinary overview that allows us to draw a picture as intriguing and fascinating as possible on the benefits of curcumin and also to suggest potential research fields still to be explored. In this vein, the review by Filardi et al. [1] is focused on reviewing the studies on the possible use of curcumin during pregnancy to prevent and/or reduce pregnancy-related complications, such as gestational diabetes mellitus, hypertension, and preeclampsia. Unfortunately, the information currently available on these issues is still limited and fragmentary and mainly coming from in vitro and animal studies. However, results from these studies, together with the knowledge about the hypoglycemic and anti-inflammatory effects already gathered, suggest possible positive effects of curcumin consumption during gestation, when the mother and the fetus undergo significant physiological immune-metabolic alterations that may have consequences on both maternal and fetal tissues. On the other hand, the authors also highlight that curcumin has been demonstrated to negatively affect the blastocyst stage, implantation and post-implantation embryo development in healthy animals; thus, the use of curcumin in pregnancy must be carefully evaluated and it should be avoided as self-medication until further studies are carried out to finally define the real safety and effectiveness during pregnancy.

As regards the protective effect of curcumin against T2D and insulin resistance occurrence, the paper by Thota et al. [2] reports data from a case–control study carried out in subjects at high risk of developing T2D. The group receiving curcumin supplementation was characterized by lower plasma insulin level with respect to the placebo group, and a similar trend also in the HOMA2-IR parameter indicating a better glucose control. It is worth noting that the authors showed an interesting amelioration of two parameters associated with insulin resistance, i.e., glycogenosynthase kinase-3β and islet amyloid peptide. The study, although preliminary and carried out on a small sample size, suggests a potential mechanism of action through which curcumin supplementation might exert an adjuvant activity against T2D risk factors. The therapeutic effects of curcumin on glycemic and lipid profile in T2D were further reviewed by Altobelli et al. [3] This systematic review and meta-analysis shows a significant reduction in glycosylated hemoglobin, HOMA, and LDL together with a general improvement of glucose metabolism in uncomplicated T2D patients treated with curcumin.

Among the biological activities exerted by curcumin, the anti-oxidative and anti-inflammatory properties, reported in many studies, make it a potentially effective tool in preventing and counteracting chronic-degenerative diseases, very often associated with obesity and aging, such as cardiovascular diseases, T2D, metabolic dysfunctions, neurodegenerative diseases, and cancer, all of them characterized by the presence of oxidative and inflammatory processes. 

In this regard, data are available on the anti-inflammatory activities of curcumin from preclinical and clinical studies addressed not only to demonstrate the protective effect of curcumin administration in patients suffering from pathologies, such as neurodegenerative diseases, metabolic diseases and cancer, but also to shed light on the molecular mechanisms responsible for these effects. Curcumin has been demonstrated to be capable of modulating signaling pathways, regulating relevant cellular activities and resulting in a modulation of inflammatory and oxidative processes. In particular, a downregulation of pro-inflammatory transcription factors, such as NF-κB, together with the increase in the activity of those regulating the antioxidant defense system, such as Nrf2, have been elucidated in in vitro and animal models. These biomolecular activities are most likely responsible for the modulation of inflammatory biomarkers, e.g., pro/anti-inflammatory cytokines and inflammasome, and enzymatic activities, e.g., COX2 and glutathione-related enzymes, detected in humans after consumption of curcumin by diet or supplements, which may be responsible for the decreased systemic inflammation detected at systemic level in patients suffering from several pathologies. Special attention is given to the beneficial effects curcumin may exert in obese patients. Obesity is recognized as a major risk factor for almost all non-communicable diseases, including cardiovascular diseases, type 2 diabetes, neurodegenerative diseases and some types of cancer, and represents a major health problem as its prevalence is consistently increasing all around the world. Obesity is characterized by the expansion of fat depots and the dysfunction of adipose tissue leading to altered secretory activities that induce a systemic low-grade chronic inflammation, that play a pivotal role in the pathogenesis of obesity and its clinical complications. Varì et al. [4] review the most recent studies carried out in humans, providing evidence for the role of curcumin in targeting specific molecular pathways that appear dysregulated in obesity. In addition to a number of studies demonstrating that the administration of curcumin decreases circulating inflammatory markers in overweight and obese patients, several data are reported that suggest an activity of curcumin in modulating specific factors such as adiponectin and leptin, relevant adipokines secreted by the adipose tissue that regulate the functions of organs and systems such as the brain, liver, and immune system. In overweight or obese people, these adipokines are imbalanced, leading to alterations of metabolic and inflammatory pathways. Curcumin has been shown to be able to restore the normal function of these adipokines, particularly of adiponectin, which plays a central role in the regulation of the metabolism and immune functions. It is worth noticing that a number of studies have been carried out to specifically evaluate the molecular mechanisms targeted by curcumin in adipocytes, unravelling its ability to modulate specific kinase and transcription factors responsible, in turn, for the regulation of cellular activities.

Metabolic dysfunction that is associated with oxidative stress and inflammation may greatly accelerate the onset and worsen the progression of cognitive dysfunctions by promoting brain ageing and reducing lifespan. Berry et al. [5] integrate current knowledge on the mechanisms underlying cognitive decline, highlighting the potential role of curcumin in this highly disabling and prevalent condition in the aging population which greatly affects physical health and quality of life. Natural antioxidant agents such as curcumin have pleiotropic protective effects and appear ideal to prevent or treat conditions such as Alzheimer’s disease or cognitive decline, whose origin is multifactorial. Preclinical animal models indicate a main effect of curcumin on cognitive function also thanks to its anti-inflammatory and antioxidant properties. While preclinical studies are mostly all in favor of a positive effect of curcumin in counteracting cognitive decline and age-associated brain dysfunction, clinical studies report discordant effects, highlighting the difficulty in translating basic research to the clinic. 

Results of published clinical studies, however, show promise for curcumin’s use as a therapeutic for cognitive decline. Kuszewski et al. [6] in this issue, provide another example of positive curcumin effects on brain function. They conducted an exploratory analysis of the effects of curcumin and fish oil supplementation on mental wellbeing in middle-aged and older adults. Previous studies had already indicated that curcumin supplementation may significantly reduce fatigue and enhance mood in non-depressed older adults. In this study, curcumin was found to improve vigor, compared to placebo, and reduced subjective memory complaints. However, combining curcumin with fish oil did not result in additive effects. This exploratory analysis indicates that regular supplementation with either curcumin or fish oil (limited to APOE4 non-carriers) has the potential to improve some aspects of mental wellbeing in association with better quality of life.

Curcumin is not only considered for preventive purposes, but also for therapeutic purposes in cancer therapy, which requires a killing effect on cancer cells. Wong et al. [7] further indicate the potential for curcumin in modulating the core pathways involved in glioblastoma cell proliferation, apoptosis, cell cycle arrest, autophagy, paraptosis, oxidative stress, and tumor cell motility. Glioblastoma is the most common and aggressive form of malignant primary adult brain tumor. In their review, the authors discuss curcumin’s anticancer mechanism through modulation of Rb, p53, MAPK, P13K/Akt, JAK/STAT, Shh, and NF-κB pathways, which are commonly involved and dysregulated in preclinical and clinical glioblastoma models providing the rationale for future studies. Curcumin is also well known for its potential role in inhibiting cancer by targeting epigenetic machinery, affecting DNA methylation. Fabianowska-Majewska et al. [8] review evidence for curcumin to modulate epigenetic events that are dysregulated in cancer cells and possess the potential to prevent cancer or enhance the effects of conventional anti-cancer therapy. More in detail, the review discusses the potential epigenetic mechanisms of curcumin in reversing altered patterns of DNA methylation in breast cancer, which is the most commonly diagnosed cancer and the leading cause of cancer death among women worldwide.

One main aspect that needs to be addressed by future research is the low bioavailability of curcumin. This is clearly addressed by Scazzocchio et al. [9], who explore the metabolic bases of the chemical instability and the poor detection of curcumin in plasma and urine. This could be also due to the frequent lack of curcumin derivative detection that may lead, therefore, to an underestimation of its absorption. 

However, great efforts have been made in recent years to improve curcumin’s bioavailability by addressing these various mechanisms. To overcome the low bioavailability of curcumin due to its insolubility in water, Beltzig et al. [10] administered curcumin in different water-soluble formulations, including liposomes or embedded into nanoscaled micelles. These authors successfully demonstrate that the effective concentration on different cell lines, including primary cells, was far above the curcumin concentration that can be achieved systemically in vivo, leading the authors to conclude that native curcumin and curcumin administered as food supplement in a micellar formulation are not cytotoxic/genotoxic, indicating a wide margin of safety opening new avenues in therapeutic strategies.

The review by Scazzocchio et al. [9] also highlights the influence of intestinal microbiota on the expression of curcumin effects. A reciprocal influence exists, in fact, between microbiota and curcumin, with curcumin being able to shape the composition of microbiota that, in turn, influences curcumin absorption and activity by producing a number of metabolites showing different degrees of biological activity. From this point of view, thus, what takes place in the gastrointestinal tract after curcumin ingestion can help to explain, at least partially, the paradox of the very low bioavailability of curcumin and its wide effect on health.

In the review by Marzena Jabczyk et al. [11], the data on the relationship between curcumin and microbiota are further expanded, considering the association between curcumin-induced changes in gut microbiota and attenuation of a number of diseases. The modulation of gut microbiota induced by curcumin treatment seems to be associated with an improvement of liver diseases, such as non-alcoholic fatty liver, as well as of metabolic health restoring a beneficial microbiota profile that is found altered in the course of metabolic disorders such as obesity and diabetes. Interestingly, the authors also reported data on the association of curcumin consumption, gut microbiota modifications, and amelioration of exercise performance in mice. Finally, studies on the inhibitory effect of curcumin on metabolism and proliferation of *Streptococcus mutans* in oral microbiota appear to be of some relevance as this bacterium is considered a main etio-pathological agent of dental caries.

Because curcumin preferentially accumulates in the gastrointestinal tract after oral administration, it is reasonable to hypothesize that this polyphenol may exert some influence in this district. Curcumin, indeed, not only modifies the composition of the microbiota but might also enhance the function of the intestinal barrier. This would have a significant role in the prevention and/or therapy of a number of intestinal inflammatory pathologies, the Inflammatory Bowel Diseases (IBD), characterized by exacerbated inflammatory processes due to alteration of the intestinal barrier integrity. The review by M. Roque Coelho et al. [12] highlights the effectiveness of curcumin as complementary therapy for ulcerative colitis. Unfortunately, there were only six studies that met the eligibility criteria to enter in the systematic review; however, almost all of them showed positive outcomes after the intervention with curcumin, and only one reported some mild side effects. In conclusion, the reported findings suggest that curcumin may be a safe, effective co-adjuvant in the therapy for ulcerative colitis, also inducing symptom remission.

The main concerns that arise from the evaluation of the preventive/therapeutic effects of curcumin, are, first of all, the still-small number of randomized placebo-control studies available; in addition, generally, small sample sizes have been considered, different protocols and different formulations as well as different routes of administration of curcumin used. Thus, it is quite difficult to compare the results and to provide a standard protocol for the use of this promising natural compound. For these reasons, and taking into account the growing use of curcumin by the general population, further investigations to confirm and expand current findings are mandatory and research on curcumin and its effects on human health have to be fostered and promoted.
